# Effect of Cardiac Phase on Cardiac Output Index Derived from Dynamic CT Myocardial Perfusion Imaging

**DOI:** 10.3390/tomography8020092

**Published:** 2022-04-14

**Authors:** Sergio C. H. Dempsey, Ting-Yim Lee, Abbas Samani, Aaron So

**Affiliations:** 1 School of Biomedical Engineering, Western University, London, ON N6A 3K7, Canada; sdempse2@uwo.ca (S.C.H.D.); asamani@uwo.ca (A.S.); 2 Department of Medical Biophysics, Western University, London, ON N6A 3K7, Canada; tlee@robarts.ca; 3 Imaging Program, Lawson Health Research Institute, London, ON N6C 2R5, Canada; 4 Imaging Research Laboratories, Robarts Research Institute, London, ON N6A 5B7, Canada; 5 Department of Electrical and Computer Engineering, Western University, London, ON N6A 3K7, Canada

**Keywords:** cardiac output index, computed tomography, myocardial perfusion imaging, time-enhancement curve

## Abstract

**Purpose:** The aortic time-enhancement curve obtained from dynamic CT myocardial perfusion imaging can be used to derive the cardiac output (CO) index based on the indicator dilution principle. The objective of this study was to investigate the effect of cardiac phase at which CT myocardial perfusion imaging is triggered on the CO index measurement with this approach. **Methods:** Electrocardiogram (ECG) gated myocardial perfusion imaging was performed on farm pigs with consecutive cardiac axial scans using a large-coverage CT scanner (Revolution, GE Healthcare) after intravenous contrast administration. Multiple sets of dynamic contrast-enhanced (DCE) cardiac images were reconstructed retrospectively from 30% to 80% R-R intervals with a 5% phase increment. The time-enhancement curve sampled from above the aortic orifice in each DCE image set was fitted with a modified gamma variate function (MGVF). The fitted curve was then normalized to the baseline data point unaffected by the streak artifact emanating from the contrast solution in the right heart chamber. The Stewart–Hamilton equation was used to calculate the CO index based on the integral of the fitted normalized aortic curve, and the results were compared among different cardiac phases. **Results:** The aortic time-enhancement curves sampled at different cardiac phases were different from each other, especially in the baseline portion of the curve where the effect of streak artifact was prominent. After properly normalizing and denoising with a MGVF, the integrals of the aortic curve were minimally different among cardiac phases (0.228 ± 0.001 Hounsfield Unit × second). The corresponding mean CO index was 4.031 ± 0.028 L/min. There were no statistical differences in either the integral of the aortic curve or CO index among different cardiac phases (*p* > 0.05 for all phases).

## 1. Introduction

Cardiac output (CO) describes the volume of blood being pumped out by the heart per unit of time. It is a useful metric to evaluate the cardiac function for diagnostic and prognostic purposes [1,2,3]. Several methods have been proposed for non-invasive assessment of CO, one of which is the indicator dilution method. This approach requires sampling of the aortic time–concentration curve following an intravenous bolus injection of tracers [4]. More recently, the indicator dilution method has been applied for deriving the CO index with computed tomography (CT) [5,6,7,8,9,10]. With the availability of large-coverage clinical CT scanners [11], it is feasible to assess the CO index in conjunction with myocardial perfusion from a single sequence of contrast administration and dynamic imaging of the heart.

In a quantitative CT myocardial perfusion study, a bolus of iodinated contrast solution is injected intravenously before the heart is imaged at a specific cardiac phase over a consecutive cardiac cycle. The temporal change of signal intensity in the aorta and myocardium in this short duration are used to derive myocardial blood flow. There is currently no consensus on the cardiac phase at which CT myocardial perfusion imaging is executed. Previous perfusion studies performed dynamic CT acquisitions at cardiac phases ranging from end-systole to end-diastole [12,13]. Since the temporal information of a CT image is dependent on the duration where raw scan data is collected, the shape of a CT-measured aortic time–concentration curve is dependent on the cardiac phase at which the dynamic acquisition is performed. The effect of cardiac phase on the CO index derived from CT myocardial perfusion imaging has not been elucidated.

The primary objective of this study was to examine the effect of the cardiac phase at which dynamic contrast-enhanced (DCE) cardiac CT images are obtained with a single-source large-coverage scanner on the assessment of CO index with the indicator dilution principle. The secondary objective was to investigate if the CO index derived from DCE CT cardiac images using the indicator dilution method was significantly different from that obtained with the ventricular delineation method, which is the more frequently used approach in clinical cardiac CT studies [14].

## 2. Methods

### 2.1. Study Subject

A total of 17 CT myocardial perfusion imaging studies were acquired in four farm pigs (59.87 ± 14.62 kg). Each pig had myocardial infarct induced for other research purposes [15]. Each pig was imaged two to four times with each imaging session on the same pig separated by at least three weeks. Due to the limited number of pigs available for this investigation, each imaging study was treated as an independent study subject to maximize the number of samples (17 instead of 4) for statistical analysis. This assumption was because the primary study objective was to evaluate the effect of cardiac phase at which CT image acquisition and reconstruction were performed on the CO index derived from the indicator dilution method. To further support this assumption, a two-way repeated measure ANOVA test was performed on three of the four pigs, which had DCE cardiac images acquired at baseline, 1-week and 4-week post infarction to evaluate if temporal progression of myocardial injury influenced the assessment of CO index, along with the potential effect of cardiac phase. The ANOVA test confirmed no statistically significant effect on the assessment of CO index from the day of imaging (*p* > 0.05). The mean heart rate of the 17 CT myocardial perfusion studies was 60.68 ± 7.79 beats per minute.

### 2.2. Dynamic CT Acquisition and Image Reconstruction

In each imaging study, the pig was anesthetized and mechanically ventilated in a supine position on the scanner table, before a bolus of iodinated contrast solution (Isovue 370, 0.7 mL per kilogram body weight) was intravenously injected into the left front leg at 4 mL/s followed by a saline flush at the same injection rate. This injection site facilitated the contrast solution to reach the heart with the shortest path. A total of 22 cardiac axial scans covering the heart were acquired with a GE Healthcare Revolution CT scanner (Waukesha, WI, USA) at 100 kV tube voltage, 100 mA tube current, 280 ms gantry rotation speed and 16 cm axial coverage. During the dynamic acquisition, the electrocardiogram (ECG) of the pig was simultaneously recorded and the ventilator was turned off to minimize respiratory motion. After the acquisition was completed, dynamic contrast-enhanced (DCE) cardiac images were retrospectively reconstructed with adaptive statistical iterative reconstruction (ASiR, 100% setting) at 30% to 80% of the R-R interval with a 5% phase increment according to the recorded ECG signals. The images reconstructed at the 30% and 80% R-R intervals exhibited the smallest and largest cross-sectional area of the contrast-filled left ventricle, respectively, confirming that these cardiac images provided a good approximation of the end-systolic and end-diastolic phases.

### 2.3. CT Derivation of CO Index

#### 2.3.1. Implementation of Indicator Dilution Method

The CO index can be assessed using the Stewart–Hamilton (S–H) equation (Equation (1)) [16], which states that the volumetric flow rate of blood through an artery can be measured by sampling the concentration of an indicator (tracer) injected into the blood stream upstream from the artery over time. The sampling site should be at a distance downstream from the injection site to ensure uniform mixing of the tracer with blood. These conditions for assessing the CO index were satisfied by injecting into a vein and sampling at the root of the aorta (above the orifice of the ascending aorta). Specifically, the CO index or volumetric flow rate through the aorta equals the ratio of the mass of injected indicator (m) to the integral of the aortic time–concentration curve during the first pass, C_a_(t):

(1) $$\mathrm{CO}=\frac{m}{\int^{\;}C_{a}\left(t\right)\mathrm{dt}}$$

The indicator used in our CT myocardial perfusion studies was iodine-based contrast solution (Isovue 370, Bracco Imaging, Milan, Italy). The concentration of iodine per unit volume (milligram per milliliter or mg/mL) in a CT image pixel can be estimated according to the linear relationship between increase in CT number (enhancement) and iodine concentration. In a CT perfusion study, a sequence of axial CT scans is acquired after bolus injection of contrast solution to assess the temporal change of iodine concentration in a region of interest. Hence, the denominator in Equation (1) can be obtained in the form of a time-enhancement curve via dynamic CT acquisition. The recirculation phase of a sampled time-enhancement curve can be removed by extrapolating the end of the washout phase during the first pass to the baseline. Integration of the extrapolated aortic time-enhancement curve, along with the injected iodine mass in milligram (mg) units estimated from the applied concentration and volume of contrast solution, allows CO to be estimated in liter units per minute (L·min^−1^).

#### 2.3.2. Sampling Location and Unit Conversion

Each aortic time-enhancement curve was sampled at a region approximately 10 mm above the aortic orifice to estimate the volume of blood being pumped out of the heart per unit of time. The sampling region was selected by concomitantly reviewing the DCE images reformatted into the three orthogonal views (short axis, horizontal and vertical long axis). The integral of aortic time-enhancement curve sampled from a series of DCE CT images had a unit of “Hounsfield Unit × second” or “HU·s”. In our studies, the CT perfusion scans were acquired with a 100 kilovolt (kV) tube voltage, and a conversion factor of 25 HU per mg/mL was used to convert the mass of iodine into “(mg/mL)·s” or “(mg/L)·min” [17]. Substituting the integral and mass of iodine expressed in the above-mentioned units into Equation (1) gave the CO index in units of L·min^−1^.

#### 2.3.3. Smoothing and Normalization of Sampled Time-Enhancement Curve

**Figure 1:**
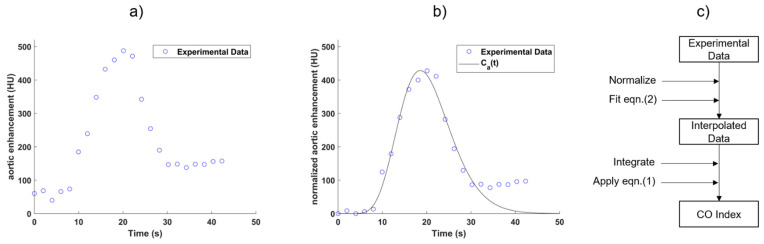
(**a**) The temporal change in aortic enhancement was monitored from dynamic CT imaging after an intravenous bolus injection of iodinated contrast solution. (**b**) The aortic time-enhancement curve C_a_(t) was obtained by fitting the experimental data acquired from CT with a modified gamma variate function (MGVF) followed by baseline subtraction. (**c**) Schematic illustration of derivation of the CO index from the experimental data and C_a_(t).

Due to the presence of image noise arising mainly from X-ray photon statistics [18], any sampled time-enhancement curve was denoised before deriving the CO index with the S–H equation. Several methods based on the cardiovascular physiology were proposed to obtain an idealized bolus curve at the sampling site in response to a bolus input of tracers [19,20,21]. Some methods treated the function C_a_(t) (the denominator in Equation (1)) as the convolution of a transfer function with a probability density function [19,20], while other methods suggested the shape of C_a_(t) could be described by a modified gamma variate function (MGVF) [16], as shown in Equation (2):

(2) $$C_{a}\left(t\right)=K{\left(t-\mathrm{AT}\right)}^{\alpha }e^{-\left(t-\mathrm{AT}\right)/\beta }$$

where K,  α, and β are constants, AT is the arrival time of the tracers relative to the injection time. The MGVF assumes that the cardiovascular system is a series of mixing compartments [22] and is an attractive approach due to its simplicity and has been applied to estimate the CO index in clinical studies [23]. Fitting of the sampled data points with a MGVF was performed using a least squares approach, with the data points in the recirculation phase excluded for fitting, and the gamma variate fitting was extrapolated to less than 1% of the maximum amplitude of the curve. Figure 1 illustrates an aortic time-enhancement curve acquired from dynamic CT acquisition and fitted with a MGVF.

The aortic time-enhancement curve has a non-zero baseline (from the inherent CT density of blood) that must be subtracted for the calculation of CO index using Equation (1). The baseline-subtracted sampled bolus curve is usually obtained by subtracting the mean baseline value from the raw sampled bolus curve. As an example, Figure 1a shows the CT numbers in the ascending aorta after an intravenous bolus injection of contrast solution. In this example, the number of baseline data points in the series was five, since there was no sharp increase in signal intensity until the sixth data point. The mean baseline CT number in the ascending aorta was calculated by averaging the first five data points and was found to be approximately 60 HU. This baseline CT number was subtracted from each subsequent data point in the series for baseline normalization (see Figure 1b).

**Figure 2:**
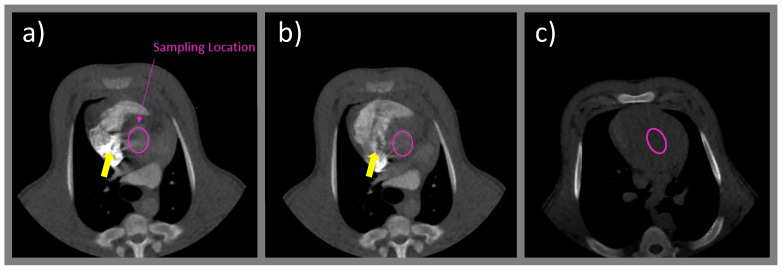
Streak artifacts (yellow arrows) emanating from highly attenuating contrast solution in the right atrium at the (**a**) end-systolic and (**b**) end-diastolic phases during the initial stage of the first pass. The artifacts spilled over into the adjacent ascending aorta (pink elliptical region), where the aortic time-enhancement curve was sampled for assessment of the CO index. The two proposed baseline subtraction approaches were evaluated against the reference baseline value measured from the middle section of the left ventricle prior to contrast arrival (**c**).

At the initial stage of the first pass, however, the streak artifacts emanating from highly attenuating contrast solution in the right heart chamber (especially the right atrium) distorted some of the baseline data points, which could subsequently lead to overestimation or underestimation of the mean baseline value (Figure 2). To overcome this issue, a method was used by selecting only the baseline data point(s) that was/were not affected by streaks for the curve normalization.

In each pig, the mean CT number measured in the middle section of the left ventricle without contrast filling was used as the reference baseline value to determine the discrepancy in the baseline of the aortic time-enhancement curve determined with the two normalization approaches—single data point vs. average of multiple data points. The mean squared error (MSE) was used to evaluate the magnitude of discrepancy from the reference baseline value.

### 2.4. Derivation of CO Index at Different Cardiac Phases

From each CT perfusion study, a total of 11 DCE cardiac image sets were reconstructed from 30% to 80% R-R intervals with a 5% increment in phase. As there were 17 CT myocardial perfusion studies, a total of 187 aortic time-enhancement curves were generated for the assessment of CO index with the indicator dilution principle. As discussed in Section 2.3, the CO index depends on both the mass of iodine and the integral of the aortic time-enhancement curve acquired from dynamic CT acquisition. Since the contrast solution was injected by a powered injection pump with exact concentration and volume programmed, the variability in CO measurement among different cardiac phases would be contributed entirely from the differences in the aortic time-enhancement curve sampled among these phases. Hence, the integral of the fitted normalized aortic time-enhancement curve along with the CO index derived from it were compared among different cardiac phases using one-way ANOVA.

### 2.5. Derivation of CO Index with Ventricular Delineation Method

**Figure 3:**
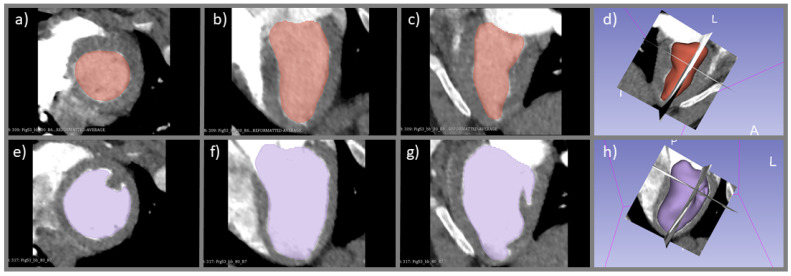
Segmentation of the left ventricular blood pool in the short axis (**a**,**e**), horizontal long axis (**b**,**f**), and vertical long axis (**c**,**g**) at the end-systolic (top row) and end-diastolic (bottom row) phases for assessment of the CO index with the ventricular delineation method. The relative position of the three orthogonal planes at the end-systolic and end-diastolic phases are illustrated in (**d**,**h**), respectively.

CO was also measured using the ventricular delineation method to compare with the indicator dilution method. The ventricular delineation method is based on the following equation:

(3) CO=(EDV−ESV)×HR

where EDV and ESV were the end-diastolic volume and end-systolic volume, respectively. HR was the heart rate recorded by the ECG monitor. From each DCE image set covering the whole heart, the images with maximum enhancement in the left ventricular blood pool were selected to measure the EDV and ESV using the software 3D slicer [24] (Figure 3). After carefully reviewing all DCE image sets, 30% and 80% of the R-R intervals were selected to represent the end-systolic and end-diastolic phases, respectively. With a 3D slicer, the blood pool in the left ventricle was first automatically segmented with a predefined CT number threshold, and the boundary of the papillary muscles were then manually adjusted if needed to minimize the overestimation or underestimation of the blood pool volume. A planar cut through the atrioventricular junction at the mitral valve was used to segment between the left atrium and left ventricle. The CO index derived from the ventricular delineation method and the indicator dilution method at end-diastole (80% R-R interval) for each study subject were compared using a paired *t*-test.

## 3. Results

### 3.1. Smoothing and Normalization of Aortic Time-Enhancement Curve Sampled from Dynamic CT Acquisition

**Figure 4:**
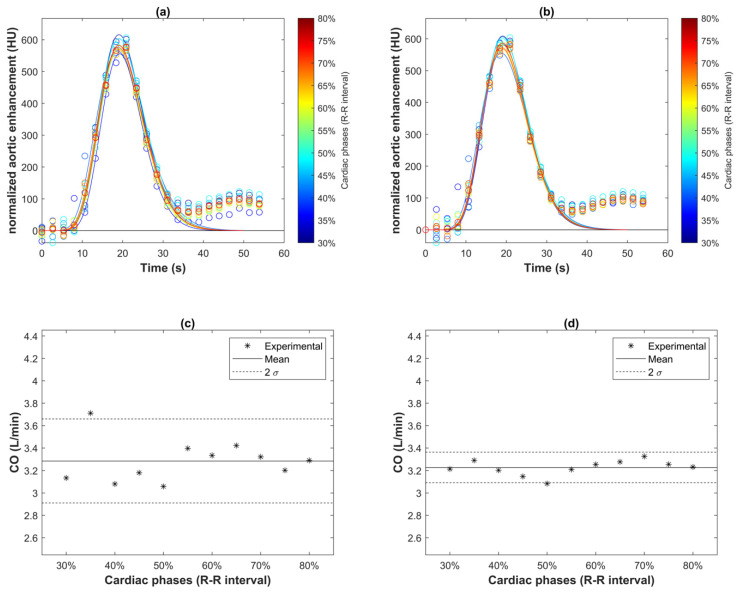
Two curve normalization approaches for assessment of CO index in a pig study—baseline subtraction using (**a**) the averaged value of all baseline data points and (**b**) only the baseline data point unaffected by streak artifacts. The curves shown in (**a**,**b**) were acquired from a pig at different cardiac phases, ranging from 30% to 80% of the R-R interval, with each cardiac phase represented by a color illustrated in the color-coded bar adjacent to the graphs. The estimated CO indexes corresponding to the two curve normalization approaches are shown in (**c**,**d**), respectively. The CO indexes estimated with the second normalization approach among different cardiac phases were less deviated from each other.

The MSE corresponding to the single baseline point normalization was 27.2 HU. In contrast, the MSE corresponding to the average baseline point normalization was 38.2 HU. The results indicate that using only the baseline data point unaffected by streak artifacts leads to a smaller discrepancy in the baseline estimation for the sampled aortic time-enhancement curve, which may sequentially result in a more accurate estimation of the area under the curve and CO index. Figure 4a,b compares the MGVF-fitted normalized aortic curves associated with the two baseline normalization approaches. These figures confirm that the curve normalization to a single unaffected baseline point led to less deviation among different cardiac phases. The corresponding CO indexes associated with the two normalization approaches were compared in Figure 4c,d. The derived CO index was less fluctuated among different cardiac phases when the single baseline point normalization approach was used.

### 3.2. Effect of Cardiac Phase on CO Index Derived from Indicator Dilution Method

**Figure 5:**
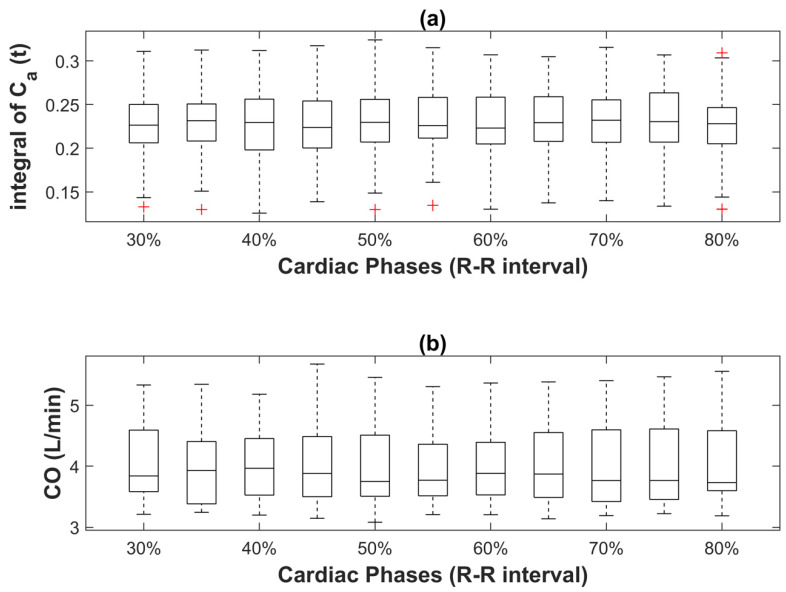
(**a**) Integrals of the fitted normalized aortic time-enhancement curves at different cardiac phases in 17 CT myocardial perfusion studies. Curve fitting was performed by using a modified gamma variate function (MVGF) and the fitted curve was normalized to a single baseline point unaffected by streak artifacts. The crosses in the boxplot represent outliers. Both the integrals and the corresponding CO indexes shown in (**b**) were not statistically different among different cardiac phases.

The boxplots in Figure 5 depict the integral of the aortic time-enhancement curve and the CO index in the 17 CT myocardial perfusion studies at each cardiac phase using the single baseline point normalization and indicator dilution method. The mean and standard deviation of the integral of the aortic curve among different cardiac phases were 0.228 ± 0.001 HU·s. The mean and standard deviation of the CO index among different cardiac phases were 4.031 ± 0.028 L·min^−1^. There were no statistically significant differences in either the integral of aortic curve or CO index among different cardiac phases (*p* > 0.05 for all cardiac phases).

### 3.3. Difference in CO Index Derived from Indicator Dilution and Ventricular Delineation Methods

**Figure 6:**
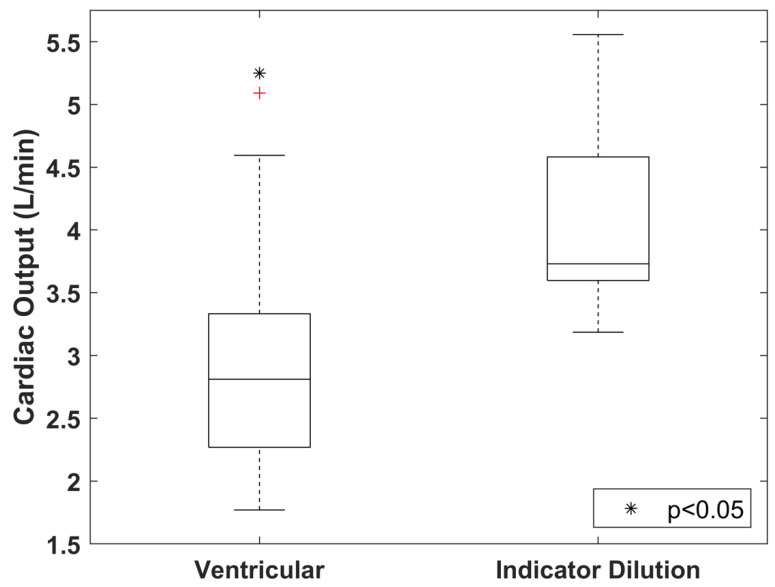
Comparison in the CO indexes assessed with the ventricular delineation and indicator dilution methods in 17 CT myocardial perfusion studies. The cross in the boxplot represents outliers; * denotes *p* < 0.05 with respect to the indicator dilution method.

The CO index assessed by the ventricular delineation method (Figure 3) in 17 CT perfusion studies were 2.96 ± 0.90 L·min^−1^, and the difference with that derived by the indicator dilution method (4.06 ± 0.74 L·min^−1^) reached statistical significance (*p* < 0.05, Figure 6). On average, the CO index estimated with the ventricular delineation method was approximately 73% of that derived with the indicator dilution method. The figure of merit (ratio of standard deviation to mean) of the ventricular delineation method in the 17 CT perfusion studies was also much larger than that of the indicator dilution method (0.304 vs. 0.045), confirming a greater degree of variation in CO assessment when the ventricular delineation method was used.

## 4. Discussion

### 4.1. Smoothing and Normalization of Sampled Aortic Time-Enhancement Curve

Previous CT studies [5,6,7,8,9,10] that used the indicator dilution method to assess CO did not explicitly discuss the curve normalization strategy. The results of our studies showed that normalizing the sampled aortic time-enhancement curve to its mean baseline value, estimated by averaging the first several non-enhanced data points, resulted in a greater variation in the normalized curve, and sequentially, a greater variability in the CO index derived from the S-H equation among different cardiac phases. In contrast, a smaller variation in the normalized curve among different cardiac phases was achieved by normalizing the sampled aortic time-enhancement curve to only the non-contrast data point unaffected by streak artifacts. The smaller fluctuation in normalized aortic time-enhancement curve resulted in a more robust curve-smoothing with the MGVF, which in turn led to a smaller discrepancy in the derivation of the CO index using the S-H equation at different cardiac phases.

### 4.2. Effect of Cardiac Phase on CO Index Derived from Indicator Dilution Method

Figure 4a,b revealed that the data outliers in each sampled aortic time-enhancement curve corresponded to the beginning of the imaging session, where the concentration of the iodinated contrast solution in the right atrium was at the peak. The rapid circulation of contrast solution during each gantry rotation in a dynamic acquisition sequence led to inconsistency in X-ray attenuation among different projection paths. Cardiac motion contributed to additional mixing of the contrast solution in the heart, which might exacerbate the swirling streak artifacts (in the form of hypo- and hyper-enhanced lines) in DCE images and resulted in more fluctuation in the sampled aortic time-enhancement curve. The impact of contrast solution causing streaking and thus CT number fluctuation was mainly seen in the baseline phase of the sampled aortic curve for two reasons: first, the contrast solution in the adjacent right atrium was the most concentrated in the initial stage of the first-pass and dynamic acquisition sequence; second, the signal-to-noise ratio in the upslope and downslope of a sampled time-enhancement curve was high, making the data fluctuation arising from rapid movement of contrast solution or other factors such as beam hardening and photon noise relatively less discernible.

Our studies demonstrated a non-significant cardiac phase dependency on the CO index derived using the S-H equation. The CO assessment with the indicator dilution approach was highly reproducible across a wide range of cardiac phases, from end-systole to end-diastole (30% to 80% R-R intervals), where clinical cardiac CT perfusion studies are typically performed. The minimal fluctuation in CO measurement among these cardiac phases (approximately 0.1 L·min^−1^) facilitates the concomitant measurement of CO and myocardial perfusion with different dynamic CT myocardial perfusion imaging protocols proposed for assessing myocardial ischemia. Since the mass of tracer injected into each pig was unchanged, the variations in CO measurement among different cardiac phases were contributed entirely by the differences in the sampled aortic time-enhancement curves. 

### 4.3. Difference in CO Index Derived from Indicator Dilution and Ventricular Delineation Methods

The difference in the derivation of CO index between the indicator dilution and ventricular delineation methods was previously investigated [8,9]. Compared with our current studies, a smaller discrepancy in CO between the two methods was observed in previous studies (0.079 ± 1.22 L·min^−1^ [8] and 2.45 ± 1.28 L·min^−1^ [9] vs. 1.07 ± 0.70 L·min^−1^). However, our results should be more accurate, given the fact that a more advanced CT scanner was used, which offered a much thinner slice thickness (2.5 mm vs. 8 mm [9] and 10 mm [8]) to optimize the spatial resolution in the transaxial direction; a much faster gantry rotation speed (0.28 sec/rot vs. 0.5 sec/rot [9]), which reduced the overlapping of the left ventricular volume between the end-systole and end-diastole arising from suboptimal temporal resolution; and a larger scan coverage (16 cm vs. 4 cm [8]), which minimized the stepping artifact across the heart due to limited coverage per acquisition.

As CO describes the volume of blood being pumped out by the heart per unit time, the CO assessment obtained with the ventricular delineation method is theoretically closer to the ground truth. However, the accuracy of this method in real-world applications is limited by the temporal resolution of the CT scanner. A minimum set of projections (scan data) covering approximately 180 degrees plus the beam fan angle is required to reconstruct a CT image [25], and as such, the image reconstruction window spans over multiple cardiac phases rather than a single cardiac phase, and sequentially, the systolic and diastolic images are bound to overlap with each other to some extent, with the degree of phase overlap depending on both the gantry rotation speed and heart rate of the scanned subject. The ventricular delineation method is also more susceptible to inter- and intra-observer variability, since the separation between the left atrium and ventricle, as well as between the ventricular blood pool and papillary muscles, is not definitive. The presence of motion-induced blurring in DCE images may further reduce the reproducibility of the ventricular delineation method. Due to these limiting factors, the ventricular delineation method exhibited a larger deviation in CO measurement in the 17 CT perfusion studies. The corresponding mean value of CO index was also lower than expected, with the value of CO index in several studies less than 2 L·min^−1^, which was unreasonable for pigs with approximately 60 kg in weight (Figure 6). In contrast, the indicator dilution method was less affected by these limiting factors, leading to a more reasonable mean value of CO index with less fluctuation among the 17 CT perfusion studies.

A correction factor of 0.71 has been previously proposed to compensate for the higher CO estimated by the indicator dilution method relative to the ventricular delineation method in human studies. [9] The results of our preclinical studies suggested a similar correction factor of 0.73. More studies will help determine if a simple correction factor can be applied to the CO index derived with either approach. Furthermore, with future advances in CT technologies, the temporal resolution of CT image is expected to improve, which may reduce the discrepancy in assessment of the CO index between the ventricular delineation and indicator dilution methods.

### 4.4. Study Limitations

Although our findings suggested normalization of aortic time-enhancement curve to a single undistorted baseline data point yielded a more reproducible CO assessment among different cardiac phases, the curve normalization would have been more robust if there had been more than one undistorted baseline data point available in our image sets. However, it is difficult to predetermine the number of undistorted baseline data points available in a clinical CT perfusion study, due to various sources of artifacts. Hence, curve normalization should be optimized on a case-by-case basis. Based on our previous assessment, the image noise level of the same CT system with identical scan settings is approximately 5 HU [13], which is subtle compared to the shift in the aortic CT number (50 HU or higher) shown in our pig studies, due to the circulation of contrast solution in the right heart chamber. Furthermore, our study did not evaluate the CO assessment with the indicator dilution approach using a dual-source CT scanner. This scanner configuration has a limited axial scan coverage but is frequently used for CT myocardial perfusion imaging in clinical settings. To overcome the suboptimal sampling frequency associated with the table joggling scan mode of a dual-source CT system, the arterial time-enhancement curve can be sampled from the middle of left ventricle instead of the root of ascending aorta, with slice overlapping between the two adjacent table positions, though the robustness of this imaging approach for CO index measurement should be investigated further.

## 5. Conclusion

This study investigated whether the CO index derived from dynamic CT myocardial perfusion imaging based on the indication dilution principle was dependent on the cardiac phase at which the DCE cardiac images were acquired. Our findings showed that the derived CO index was indifferent among cardiac phases if the sampled time-enhancement curve was properly fitted with a MGVF and normalized to only the baseline data point unaffected by streak artifacts emanating from contrast solution. Compared with the ventricular delineation method, assessment of the CO index with the indicator dilution method was more reproducible among different cardiac phases.

## Data Availability

Data sharing not applicable.
